# A prediction study of warfarin individual stable dose after mechanical heart valve replacement: adaptive neural-fuzzy inference system prediction

**DOI:** 10.1186/s12893-018-0343-1

**Published:** 2018-02-15

**Authors:** Huan Tao, Qian Li, Qin Zhou, Jie Chen, Bo Fu, Jing Wang, Wenzhe Qin, Jianglong Hou, Jin Chen, Li Dong, on behalf of the CLIATHVR multicenter clinical study team

**Affiliations:** 1 Department of Evidence-based Medicine and clinical epidemiology, West China Hospital, Sichuan University, 37 Guo Xue Xiang, ChengDu, 610041 China; 2 Department of Nutrition, The Second affiliated hospital of Chongqing medical university, Chongqing, China; 3 Department of Anesthesiology, China Mianyang Central Hospital, Mianyang, China; 4 Department of Cardiovascular Surgery, Tianjin central hospital, Tianjin, China; 5 Department of Career development division, The fourth affiliated hospital of Anhui Medical University, Hefei, China; 6 Department of Social Medicine and Health Management, Shandong University, Jinan, China; 7 Department of Cardiovascular Surgery, West China Hospital, Sichuan University, Chengdu, China

**Keywords:** Rational medication, Warfarin, Heart valve surgery, Adaptive neural-fuzzy inference system, Dose prediction

## Abstract

**Background:**

It’s difficult but urgent to achieve the individualized rational medication of the warfarin, we aim to predict the individualized warfarin stable dose though the artificial intelligent Adaptive neural-fuzzy inference system (ANFIS).

**Methods:**

Our retrospective analysis based on a clinical database, involving 21,863 patients from 15 Chinese provinces who receive oral warfarin after the heart valve replacement. They were allocated into four groups: the external validation group (A group), the internal validation group (B group), training group (C group) and stratified training group (D group). We used a univariate analysis of general linear models(GLM-univariate) to select the input variables and construct two prediction models by the ANFIS with the training and stratified training group, and then verify models with two validation groups by the mean squared error(MSE), mean absolute error(MAE) and the ideal predicted percentage.

**Results:**

A total of 13,639 eligible patients were selected, including 1639 in A group, 3000 in B group, 9000 in C group, and 3192 in D group. Nine input variables were selected out and two five-layered ANFIS models were built. ANFIS model achieved the highest total ideal predicted percentage 63.7%. In the dose subgroups, all the models performed best in the intermediate-dose group with the ideal predicted percentage 82.4~ 86.4%, and the use of the stratified training group slightly increased the prediction accuracy in low-dose group by 8.8 and 5.2%, respectively.

**Conclusion:**

As a preliminary attempt, ANFIS model predicted the warfarin stable dose properly after heart valve surgery among Chinese, and also proved that Chinese need lower anticoagulation intensity INR (1.5–2.5) to warfarin by reference to the recommended INR (2.5–3.5) in the developed countries.

## Background

Warfarin is the most commonly prescribed anticoagulant with its best effectiveness and low-cost for long-term management with cardiac valve replacement [1, 2]. However, the use of warfarin is restricted by the narrow therapeutic window and highly individual variation, for instance, bleeding and embolism complications, resulting from the insufficient dose or overdose anticoagulation, led warfarin to be the main causes of severe adverse event worldwide [3, 4]. This fact is more apparent in Chinese by reference to the recommended INR(2.5–3.5) for developed countries. Therefore, the individual rational medication of warfarin dosage is the key to guarantee the safety and therapeutic effectiveness during the anticoagulation therapy.

Currently, numerous clinical and genetic prediction models have been applied to predict the warfarin stable dose in the anticoagulation therapy, most of which are based on multiple linear regression (MLR) [5–9]. To our acknowledgement, MLR has some well-known limitations in identifying and dealing with the nonlinear relationship between various influencing factors, which may cut down the prediction accuracy of the models. Hence, MLR might not be the most appropriate method to predict warfarin stable dose accurately, especially for the individuals. Adaptive Neuro-Fuzzy Inference System (ANFIS), as a new artificial intelligence, has strong skills of classification identifying complex nonlinear relationship and other possible interactions among predictors in the form of fuzzy rules quickly, and dealing with the nonlinear relationships between input variables by adaptive learning features. Previous studies have proved that ANFIS has been frequently applied in medical field successfully through establishing prediction models based on clinical database [10–15].

Hence, our study aims to predict the individual warfarin stable dose by ANFIS model based on a big clinical database retrospectively, which was generated by a registered and multicenter cohort with 21,863 cases have received the heart valve replacement in China and validate it with the internal and external validation groups.

## Methods

### Study design and setting of the study

We conducted our study retrospectively based on a database named “Chinese Low Intensity Anticoagulant Therapy after Heart Valve Replacement” (CLIATHVR), which was registry study involving 35 participating centers from 15 provinces in China collecting all patients who underwent heart valve replacement received anticoagulation with warfarin. We finally enrolled 21,863 collected cases from April 1, 2011 to November 5,2014, and authors had access to information that could identify individual participants during or after data collection after application. Written informed consent was obtained from all participants and the study was approved by the Ethics Committee of the West China hospital in Sichuan university with the number 2017(92).

### Characteristics of participants

The inclusion criteria were as follows: age above 18 years, receive oral anti-coagulation warfarin in regular and monitor INR as index after receiving cardiac valve surgery; assure the fluctuation of INR less than 0.2 units for three times continuously during the later follow-up.

The exclusion criteria: Severe liver or kidney dysfunction before or after the operation; combined use of non-steroidal anti-inflammatory drugs or other drugs affecting coagulation; anticoagulant.

complications occurred during anticoagulant therapy (thrombosis, embolism, bleeding, death result).

### Variables

#### Input variables and output variables

We identified 45 potential input variables among 706 items derived from the CLIATHVR database based on clinical experience and knowledge related to warfarin, including 16 items of demographic data; 17 items of preoperative medical examination data; 9 items of surgery type; 3 items of postoperative anticoagulation data. Due to the stability of screening the variables by the GLM-univariate analysis and the value of partial squared eta could reflect the influence of each variable, we adopted it to get the influential input variables from potential input variables. On the premise of presenting the prediction function of ANFIS models, we choose partial squared eta ≥0.002 in order to simplify the predicted models. The warfarin stable dose was set as the output variable.

#### Group setting

We constructed four groups by eligible participants: the external validation group(A group), the internal validation group(B group), the training group(C group) and the stratified training group(D group). Since all the cases were arranged by the enrollment time, we extracted the last 12% enrolled cases of the whole sample as the external validation group (A Group). The rest of the eligible patients were randomly divided into the internal validation groups(B group)and the training (C group) and by the ratio of 1:3. Meanwhile, the training group (C group)was divided into three sub-groups based on the warfarin origin: the high-dose (warfarin stable dose ≥3.125 mg/d), intermediate-dose (1.875–3.125 mg/d) and low-dose (≤1.875 mg/d) group. Given the uneven distribution in the different dose subgroups, we created a stratified training group (D group) by using the method of equal random- stratified sampling, which randomly extracted the same number of patients from the other two dose groups equal to the smallest group. The training group (C group) and stratified training group (D group)were used to train models until achieve the appropriate parameters to construct the prediction model, and the external-(A group) and internal validation groups(B group) were used to verify the prediction accuracy of the model.

### Modeling of ANFIS

The ANFIS is the achievement of a Fuzzy Inference System in the form of a multi-layer neural network where each layer is a neuro-fuzzy system component [11]. The ANFIS model was generated through Fuzzy Logic Tool box in MatlabR2010b by using the fuzzy subtractive clustering to find the fuzzy rules, which was performed by setting radii as 0.5, fis as 300, step-size as 0.01. The models were trained with the training group(C group) and the stratified training group (D group) based on the most popular learning hybrid algorithm with epochs of 1000 to adapt the parameters in the adaptive network, and the operation was stopped when the MAE was minimum. After training, the parameter of the membership was adapted to give better matching between input and output, which lead to changing the initial shape of the membership. The more changing of the membership shape before and after the training represents the most effective variables in constructing the model. The performance of the model was ascertained by the internal validation group and the external validation group as the testing and the checking data. The neural network structure contains five layers below.

- Layer 1 is the fuzz-ification layer in which each node represents a membership value to a linguistic term as a Gaussian function with the mean;
- Layer 2 provides the strength of the rule by means of multiplication operator. It performs AND operation;
- Layer 3 is the normalization layer which normalizes the strength of all rules according to the equation;
- Layer 4 is a layer of adaptive nodes. Every node in this layer computes a linear function where the function coefficients are adapted by using the error function of the multi-layer feed-forward neural network;
- Layer 5 is the output layer whose function is the summation of the net outputs of the nodes in Layer 4.

### Data measurement-model validation

Our primary outcome was the difference between predicted warfarin stable dose by ANFIS models and the actual warfarin stable dose in the clinical practice. The models’ predictive accuracy was evaluated by the external validation group(A group)and the internal validation(B group)with three indexes, including the mean squared error (MSE), mean absolute error (MAE) and the ideal predicted percentage. MAE is the average of absolute difference between the predicted dosage and the actual dosage that patients received. MSE is the square of the difference between two dosages. The ideal predicted percentage was defined as the percentage of patients whose predicted warfarin dose was within 20% of the actual dose.

### Statistical methods

Data was recorded by Microsoft Excel 2010. The comparisons of baseline characteristics of patients receiving warfarin between different groups when compared with the whole sample with the χ^2^ test for categorical variables and the independent sample *t* test for continuous variables. Difference between the predicted dose and the actual dose was analyzed by the paired t-test. All statistical tests were two-sided and a *P* value less than 0.05 was considered as statistically significant without exception. All the analyses were performed by SPSS 20.0.

## Result

### Participants and descriptive data

**Fig. 1:**
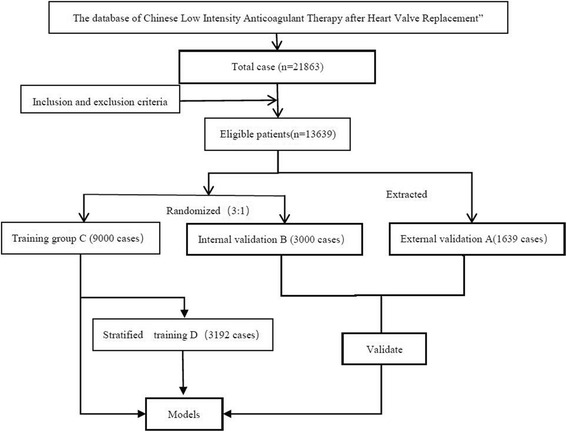
The flow diagram of our study

A total of 13,639 patients were eligible into this study, including 1639(12%) in the external validation group (A group), 3000 patients (22%) in the internal validation group (B group), 9000 patients (66%) in the training group (C group), 3192 patients in the stratified training group (D group) (the smallest number was in the low-dose group, 1064 patient were randomly drawn from the high-and intermediate-dose groups respectively). Study flow in detailed Fig. 1.

Compared with the whole sample, obvious statistical significance existed in the external validation group with the warfarin stable dose(2.54 ± 0.81 mg/d) and some basic characteristics, such as gender, height, weight and history of disease, and all the basic characteristics in the internal validation group is in accordance with the training group. The very finding complied with our design of the external validation group (A group).The main clinical characteristics of the eligible patients were described in Table 1. All the eligible patients were Chinese with 95.92% was Han, the age ranged from 18 to 92 years, and the sex ratio (F/M) was 1/0.81. The common types of surgery were mitral valve replacement (71.90%), the rest operation type including the aortic valve surgery, the tricuspid valve surgery and the mitral valve plastic. In the following anticoagulation therapy after the operation, the mean warfarin stable dose is 2.82 ± 0.89 mg/day and the main patients were concentrated in the intermediate dose group with 63.35%.

**Table 1:** the basic characteristic of the whole study

Characteristic (unit)	Total cases 13639cases N(%)	Training group 9000 cases N(%)	stratified training group 3192 cases N (%)	internal validation 3000 cases N(%)	external validation 1639 cases N(%)
Gender(female)	7538(55.27)	4897(54.41)	1750(54.82)	1688(56.27)	953(58.15)^*^
Age(year)	50.09±11.18	50.06±11.11	50.31±11.21	50.27±11.52	49.97±10.94
Nationality(Han)	13082(95.92)	8666(96.29)	3085(96.65)	2899(96.63)	1517(92.56)^**^
Height(cm)	162.67±8.06	162.89±8.08	162.72±8.03	162.68±7.96	161.38±8.00^**^
Weight(kg)	60.41±10.84	60.71±10.97	60.41±10.89	60.43±10.69	58.75±10.29^**^
BSA(m^2^)	1.61±0.17	1.62±0.17	1.61±0.17	1.61±0.17	1.58±0.17^**^
BMI(kg/ m^2^)	22.76±3.28	22.80±3.29	22.74±3.29	22.77±3.27	22.50±3.19^**^
Left ventricular diastolic diameter	56.38±13.80	56.41±13.74	56.66±13.96	56.16±14.02	56.62±13.73
Inner diameter of leftatrium(mm)	50.23±13.64	50.35±13.70	50.44±14.02	50.05±14.09	49.92±12.47
Inner diameter of right atrium(mm)	38.88±12.88	38.33±12.70	38.37±12.82	38.04±13.05	43.47±12.61^**^
EF(%)	58.60±8.92	58.56±8.88	58.55±8.67	58.99±8.91	57.56±8.78^**^
Atrial fibrillationhistory	4430(32.48)	2984(33.16)	1061(33.24)	979(32.63)	467(28.49)^**^
Cardioerter treatment	66(0.48)	51(0.57)	20(0.63)	13(0.43)	2(0.12)
Embolism history	211(1.55)	149(1.66)	60(1.88)	55(1.83)	(0.43)^**^
Thrombushistory	657(4.82)	468(5.20)	168(5.31)	126(4.20)	80(4.88)
Bleeding disease history	272(1.99)	191(2.12)	66(2.09)	81(2.70)	0(0.0)^**^
History of anticoagulant drugs	517(3.79)	349(3.88)	136(4.30)	153(5.10)	15(0.92)^**^
Hypertensionhistory	1342(9.84)	891(9.90)	322(10.18)	326(10.87)	125(7.63)^**^
Diabetes mellitushistory	428(3.14)	276(3.07)	99(3.13)	103(3.43)	49(3.00)
Operation history	1586(11.63)	1074(11.93)	380(12.02)	365(12.17)	147(8.97)^*^
ALT(IU/L)	25.86±19.81	25.90±19.66	25.63±20.06	25.55±20.17	25.25±19.96
AST(IU/L)	26.47±15.68	26.39±15.26	26.47±15.95	26.36±16.91	27.13±15.58
Total albumen(g/L)	68.64±6.69	68.80±6.65	68.90±6.63	68.63±6.76	67.75±6.74^**^
Albumin(g/L)	41.57±4.67	41.67±4.62	41.72±4.65	41.59±4.77	41.03±4.69^**^
Urea nitrogen(mmol/L)	6.13±2.09	6.17±2.10	6.18±2.10	6.10±2.06	6.03±2.08
Creatinine(μmol/L)	77.67±19.94	77.62±19.93	77.62±19.92	77.23±19.96	78.84±19.97^*^
PT(s)	13.30±4.10	13.34±4.29	13.28±4.00	13.37±3.77	12.93±3.52^**^
APPT(s)	31.69±8.50	31.91±8.33	31.85±8.33	32.16±9.52	29.61±7.04^**^
Preoperative INR	1.12±0.46	1.12±0.48	1.13±0.57	1.11±0.36	1.12±0.45
NYHA classification					^**^
I class	307(2.25)	228(2.53)	71(2.25)	70(2.33)	9(0.55)
II class	3387(24.83)	2301(25.57)	779(24.64)	728(24.27)	358(21.84)
III class	9445(69.25)	6117(67.97)	2193(69.35)	2100(70.00)	1228(74.92)
IVclass	500(3.67)	354(3.93)	149(4.71)	102(3.40)	44(2.68)
ECG					
Sinus rhythm	7880(57.78)	5201(57.79)	1816(57.43)	1769(58.97)	910(55.52)
Atrial fibrillation	5674(41.60)	3746(41.62)	1362(43.07)	1210(40.33)	718(43.81)
Atrial flutter	85(0.62)	53(0.59)	14(0.44)	21(0.70)	11(0.67)
Mitral valve surgery					
Plastic	107(0.78)	69(0.77)	31(0.98)	27(0.90)	11(0.67)
Replacement	9806(71.90)	6431(71.46)	2277(72.01)	2130(71.00)	1245(75.96)^*^
Tricuspid valve surgery					
Plastic	5071(37.18)	3262(36.24)	1141(36.08)	1066(35.53)	743(45.33)^**^
Replacement	261(1.91)	168(1.87)	65(2.06)	62(2.07)	31(1.89)
Aortic valve surgery					
Plastic	34(0.25)	22(0.24)	10(0.32)	7(0.23)	5(0.31)
Replacement	7302(53.54)	4815(53.50)	1717(54.30)	1615(53.83)	872(53.20)
Left atrial appendage occlusion method					^*^
Non-treated	12545(91.98)	8237(91.52)	2933(92.76)	2782(92.73)	1526(93.11)
Ligation	485(3.56)	330(3.67)	117(3.70)	89(2.97)	66(4.03)
Excision	130(0.95)	93(1.03)	34(1.08)	30(1.00)	7(0.43)
internal suturing	479(3.51)	340(3.78)	108(3.42)	99(3.30)	40(2.44)
Thrombus removal	1169(8.57)	788(8.76)	284(8.98)	228(7.60)	153(9.33)
Radiofrequency ablation	1157(8.48)	780(8.67)	289(9.14)	261(8.70)	116(7.08)
Left atrial volume loss	242(1.77)	168(1.87)	63(1.99)	48(1.60)	26(1.59)
Surgical bleeding	122(0.89)	82(0.91)	26(0.82)	32(1.07)	8(0.49)
Non surgical bleeding	161(1.18)	104(1.16)	38(1.20)	29(0.97)	28(1.71)
Origin of warfarin	6617(48.52)	4368(48.53)	1439(45.51)	1458(48.60)	791(48.26)
Dosage form(2. 5 mg/tablet)	1.85±0.97	1.85±0.99	1.87±1.00	1.85±1.01	1.90±0.79^*^
Starting time of anticoagulation(n days after surgery)	2.82±0.89	2.86±0.90	2.73±1.17	2.84±0.87	2.54±0.81^**^
Warfarindose-subgroup					^**^
Low-dose	1774(13.01)	1064 (11.82)	1064(33.33)	363(12.10)	347(21.17)
intermediate-dose	8640(63.35)	5702(63.36)	1064(33.33)	1905(63.50)	1033(63.03)
High-dose	3225(23.65)	2234(24.82)	1064(33.33)	732(24.40)	259(15.80)

*Abbreviations*: *BSA* Body surface area, *BMI* Body mass index, *EF* Ejection fraction, *LVDD* Left ventricular end diastolic dimension, *LAD* Left atrial diameter, *RAD* Right atrial diameter, *ALT* alanine transaminase, *AST* aspartate aminotransferase, *APTT* activated partial thromboplastin time, *INR* international normalized ratio

Note: Continuous variables materials were analyzed by using independent sample t-test, categorical data material were analyzed by using chi-square analysis. ^*^P <0.05; ^**^P <0.001. Variables :Han=0,all ethnic=1

Body surface area (BSA) =0.0061×height(cm)+0.0128×weight(kg)-0.1529; body mass index (BMI)=weight(kg)/height2 (m^2^)

### Main results

#### Input variables

We finally determined nine variables derived from the 45 potential inputs by using the GLM-Univariate (partial η^2^ ≥ 0.002)method. These nine variables were identified, including age, NYHA classification, BSA (Body surface area), RAD (Right atrial diameter), Creatinine, APPT (Activated partial thromboplastin time),Radiofrequency ablation, Warfarin origin, Starting time of anticoagulation. The most influential variables in the screening were body surface area, warfarin origin and age. All the input variables were used to construct models and the warfarin stable dose was set as the outcome variable.

#### Prediction models

**Fig. 2:**
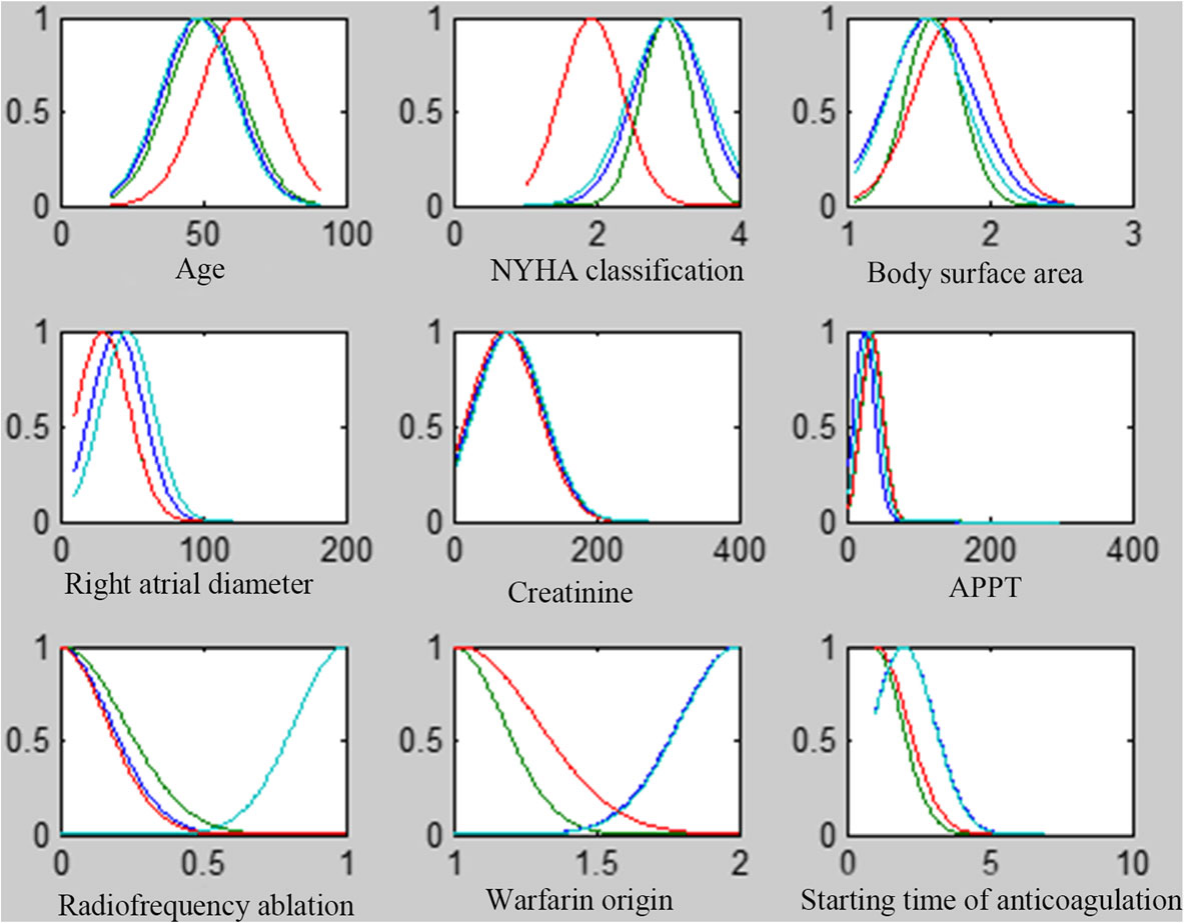
Membership function of ANFIS model

**Fig. 3:**
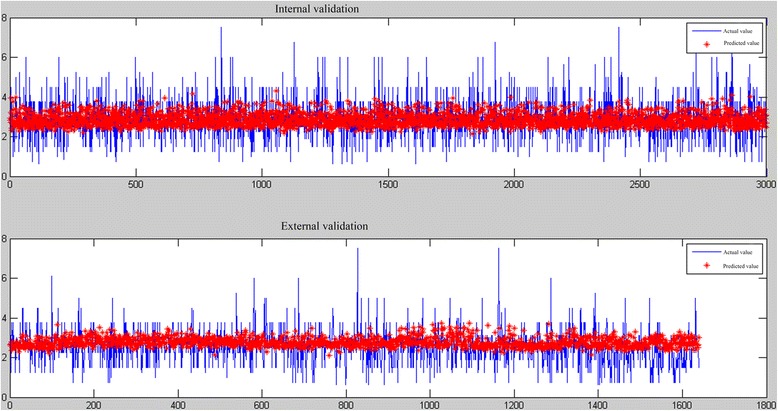
Predictive diagram of ANFIS model

**Fig. 4:**
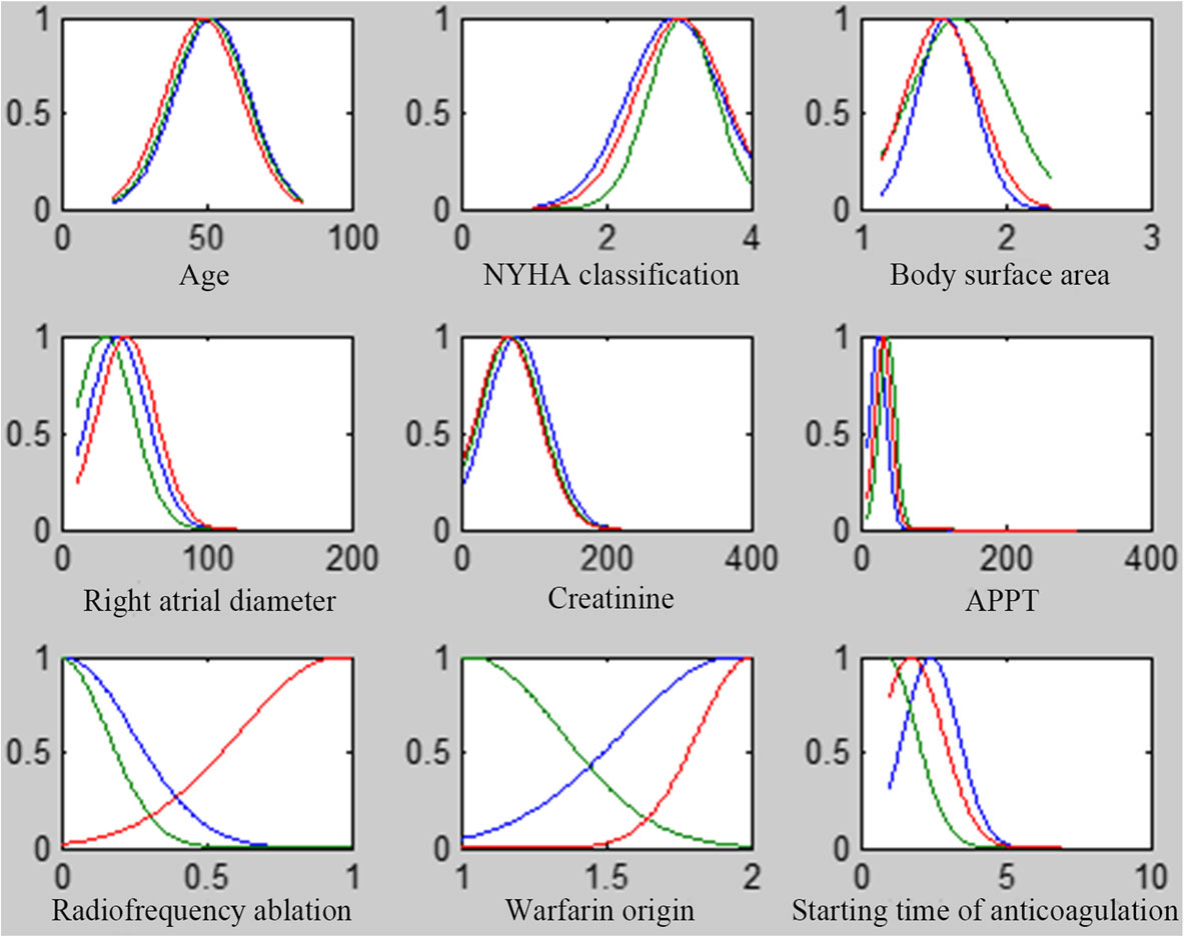
Membership function of ANFIS _stra_ model

**Fig. 5:**
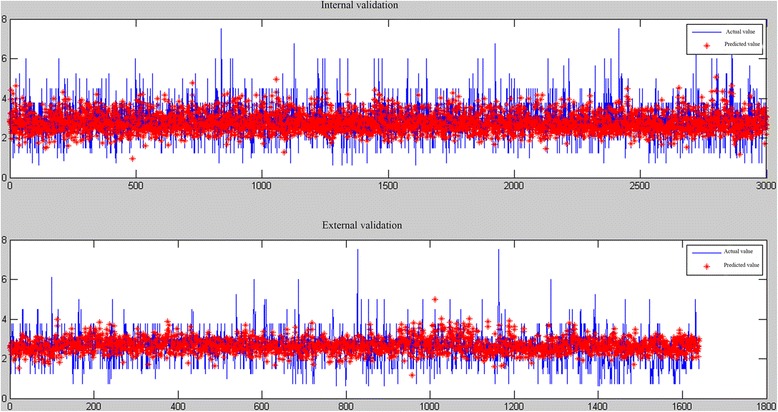
Predictive diagram of ANFIS _stra_ model

With a total of nine input variables as the input layer and the warfarin stable dose as the output layer, we constructed two five-layered ANFIS models (ANFIS model, ANFIS _stra_ model) by using the training group (C group) and the stratified group (D group)separately. The membership function and predicted results in the models were detailed in the Figs. 2, 3, 4 and 5.

#### Model validation

In the comparison of predicted value to the actual value, obvious statistical significance exists in the in the ANFIS _stra_ model while presenting no statistical significance in the ANFIS model with the highest correlation 31.3% in the internal validation group. The fact that obvious statistical significance exists in two models showed with *p* < 0.001in the external validation group showed the representative role of the external validation group (detailed in Table 2). Table 3 shows that the accuracy of the internal validation was higher than that of external validation according to the ideal predicted percentage of the internal validation (59.5%, 63.7%) and the external validation (57.0%, 60.6%). The MAE of models ranging from 0.581 mg/day to 0.621 mg/day and the MSE is less than 0.738 mg/day.

**Table 2:** Comparison of predicted value and actual value of warfarin in the validation groups

Model	Predicted value (mg/d)	Actual value (mg/d)	*P*	correlation
	*X±S*	*X±S*		coefficient
ANFIS_iv_	2.86±0.31	2.84±0.87	0.221	0.313
ANFIS_stra-iv_	2.75±0.49	2.84±0.87	0.000^*^	0.309
ANFIS_ev_	2.77±0.25	2.54±0.81	0.000^*^	0.066
ANFIS_stra-ev_	2.61±0.38	2.54±0.81	0.001^*^	0.101

The subscript “stra” in the model symbols stratified training group, “iv” symbols internal validation group and “ev” symbols external validation group. *P*-values have been obtained by Paired T test. *P*<0.05

**Table 3:** The ideal predicted percentage of warfarin in patients in the validation groups

Models	MAE(95%CI)	Under predicted percentage n (%)	Ideal predicted percentage n (%)	Over predicted percentage n (%)	MSE(95%CI)
ANFIS_iv_	0.581(0.559~0.602)	472 (15.7)	1911 (63.7)	617 (20.6)	0.688 (0.637~0.738)
ANFIS_stra-iv_	0.621 (0.600~0.643)	632 (21.1)	1786 (59.5)	582 (19.4)	0.743 (0.688~0.798)
ANFIS_ev_	0.603 (0.573~0.633)	160 (9.8)	993 (60.6)	486 (29.7)	0.739 (0.668~0.809)
ANFIS_stra-ev_	0.621 (0.592~0.650)	273 (16.7)	934 (57.0)	432 (26.4)	0.738 (0.665~0.810)

The subscript “stra” in the model symbols stratified training group, “iv” symbols internal validation group and “ev” symbols external validation group. MSE: mean squared error; MSE: mean absolute error (MAE); the ideal predicted percentage was defined as the percentage of patients whose predicted warfarin dose was within 20% of the actual dose

In order to check the clinical practicability of the models, three different dose subgroups were set detailed in Table 4, all the models performed best in the intermediate group with the ideal predicted percentage 80.1~ 86.4%. ANFIS model achieved fairly good prediction ability in the high dose group 40.2% but the ideal predicted percentage in the low dose group was poor with maximum value 9.1%. The use of stratified training group slightly increased the prediction accuracy in low dose group by 5.5–8.8%, but the reduction of the prediction accuracy either in the intermediate-dose or the high-dose group was more apparent in ANFIS models by 3.7–15.1%, especially in the high-dose group.

**Table 4:** The predictive ability of models in the different dose-subgroups

Model	Low-dose group (N%)			Intermediate -dose group (N%)			High-dose group (N%)		
	(<=1.875mg/d)			(1.875~3.125mg/d)			(>=3.125mg/d)		
	under	ideal	over	under	ideal	over	under	ideal	over
ANFIS_iv_	0 (0.0)	1 (0.3)	362 (98.7)	5 (0.2)	1645 (86.4)	255 (13.4)	467 (63.8)	265 (36.2)	0 (0.0)
ANFIS_stra-iv_	1 (0.2)	33 (9.1)	329 (90.6)	126 (6.6)	1526 (80.1)	253 (13.3)	505 (69.0)	227 (31.0)	0 (0.0)
ANFIS_ev_	0 (0.0)	0 (0.0)	347 (100.0)	5 (0.5)	889 (86.1)	139 (13.5)	155 (59.8)	104 (40.2)	0 (0.0)
ANFIS_stra-ev_	0 (0.0)	18 (5.2)	329 (94.8)	79 (7.6)	851 (82.4)	103 (10.0)	194 (74.9)	65 (25.1)	0 (0.0)

The subscript “stra” in the model symbols stratified training group, “iv” symbols external validation group

## Discussion

The study showed that the ANFIS can predict the warfarin stable dose for Chinese patients after heart valve replacement surgery properly with the total ideal predicted percentage 57.0–63.7%. In the dose subgroups analysis, all the models performed best in the intermediate group with high ideal predicted percentage 80.1–86.4%. In the high dose group, ANFIS model achieved relatively high 25.1–40.2%, but the performance of models reduced in the stratified training, especially in the high dose group.

The International Warfarin Pharmacogenetics Consortium(IWPC)in 2009 established the most famous warfarin prediction model with 4043 eligible patients [16], the clinical model was built by clinical variables, and the genetic model was built by both the clinical variables and pharmacogenetic variables, and the 1009 patients comprised the validation cohort. Our prediction accuracy of the models in the internal validation is higher than IWPC clinical model except the lower predictive accuracy in low dose group (low dose group 0.3% vs 25.9%; intermediate dose group 86.4% vs 53.6%; high dose group 36.2% vs 9.6%; the total ideal predicted percentage 63.7% vs 39.3%).When considering the number distribution of different dose group, in IWPC model, low dose group didn’t occupy the smallest part, However, it hold the smallest in our model. Hence, the number distribution can affect the predicative accuracy especially the smallest subgroup. Besides, the MAE in our models is nearly less than 4.35 mg/week which is much lower than 9.9 mg/week compared to the clinical algorithm of the IWPC. The mean warfarin stable dose in our study nearly 20 mg/week was lower than the clinical model of IWPC with the dose 28 mg/week, which was consistent with the fact that Chinese need the lower INR during the anticoagulation therapy than westerns [17, 18]. Since the mean age of the patients in this study was 50 years and less than 1% of the patients had history of embolic disorders. Compared to other western world studies, the majority of the patients in our study did not have atherosclerosis as etiological factor, those obvious differences might explain the fact that Chinese population a lower dose of warfarin is needed in comparison to those recommended in the western world. Currently, the report of database in our multicenter registered study revealed that 88.6% of the target INR was between 1.5–2.5 and the mean INR was 1.84 ± 0.53 during hospitalization. All the above findings demonstrate the fact the low intensity with the INR range from 1.5 to 2.5 might be suitable for Chinese after the heart valve surgery.

How to identify the influential variables determines the stability and the prediction performance of the model, but whether the genetic variables can benefit patients or not is still controversial. We identified nine input variables by GLM-univariate analysis and variables is not completely consistent with the IWPC models. The adding of genetic variables in the IWPC pharmacogenetic model significantly improve the predictive accuracy of the subgroup dose, but the ideal predicted percentage in the intermediate group was 54.6%, which was lower than 86.4% in our study. A multicenter, randomized, controlled trial conducted in 2013 [7] based on 455 patients concerning genotype-guided dosing of warfarin suggest that genotype-guided warfarin dosing was superior to standard dosing in the mean percentage of time(gene directs 67.4% vs the control group 60.3%), and the shorter median time needed to reach a therapeutic INR in the genotype-guided group. But another article published in the same year [19] with the same purpose indicates that the genotype-guided dosing of warfarin did not improve anticoagulation control during the first 4 weeks of therapy. Besides, the current genetic testing is expensive which isn’t in the scope of health insurance system service, and has not been popular in the developing countries. Hence, the above inconsistent results and issues would also arise if the genotype-guided dosing were implemented in the future research and clinical practice.

One important feature in our study is that we enrolled a large sample with 13,639 one single ethnic patients who received warfarin after heart valve replacement to establish and validate the models. Most previous study of warfarin prediction models based on different racial backgrounds, among which, the IWPC constructed prediction models with the maximum 4043 patients, including 55.2% White people, 30.4% Asian, 8.7% black and remaining mix population. Another important feature is the application of the internal and the external validation since that previous studies have proposed the internal validation may lead to optimism in prediction accuracy than external validation [20]. Our design of the external validation group was a feasible attempt according to obvious statistical significance among some the basic characteristics and the warfarin dose compared to the internal validation group, and our results showed that the total prediction accuracy of external validation group is lower than the internal validation group by 3.1%. Based on our previous prediction modeling research conducted in 2014 [21] and considering the linear relationship between independent variables and the dependent variable, we used the general linear models of univariate method to get the target input variables in the screening of variables.

We conducted this research rigorously, but there are still limitations to our study, one limitation was that we didn’t add the influential genetic information because of that we haven’t obtained it yet until now. In the selected input variables, the absence of major influencing factors in the difficult data collection, such as life style and drug combinations, could influence the prediction accuracy of the models by effecting the coagulation cascade. Thirdly, it is likely that some patients had more than one valve replaced, and the data we could obtained couldn’t meet our needs to do subgroup analysis. Lastly, using ANFIS, like any multidimensional analysis, importance is given to variables that have influence in that determined retrospective study. In other word, in this retrospective study, ANFIS is only a description of the phenomenon that has been analyzed, results should be validated in a prospective study in the future research before any clinical application.

## Conclusions

In conclusion, our research explored the rational medication of individualized warfarin stable dose for Chinese patients after heart valve disease treatment adapting the ANFIS with patient-related clinical parameters based on 21,863 Chinese cases. The result indicated that ANFIS could predict the warfarin stable dose properly, which might play potential guiding role for doctors in deal with the Chinese patients, and also proved that Chinese need lower anticoagulation intensity INR (1.5–2.5) to warfarin by reference to the recommended INR (2.5–3.5) in the developed countries. Our database is large and being multicentric, reinforcement learning(RL) within machine learning might be tried in the further research with this ideal scenario, and genetic database will be combined with clinical variables to predict the individualized warfarin more accurately.
